# Safety and efficacy of 0.01% and 0.1% low-dose atropine eye drop regimens for reduction of myopia progression in Danish children: a randomized clinical trial examining one-year effect and safety

**DOI:** 10.1186/s12886-023-03177-9

**Published:** 2023-10-30

**Authors:** Niklas Cyril Hansen, Anders Hvid-Hansen, Flemming Møller, Toke Bek, Dorte Ancher Larsen, Nina Jacobsen, Line Kessel

**Affiliations:** 1 Department of Ophthalmology, Copenhagen University Hospital - Rigshospitalet-Glostrup, Valdemar Hansens Vej 1-23, DK-2600 Glostrup, Denmark; 2 Department of Ophthalmology, University Hospital of Southern Denmark – Vejle Hospital, Beriderbakken 4, DK-7100 Vejle, Denmark; 3 Department of Ophthalmology, Aarhus University Hospital, Palle Juul-Jensens Boulevard 167, DK-8200 Aarhus, Denmark; 4 Department of Clinical Medicine, University of Copenhagen, Blegdamsvej 3b 33.5, DK-2200 Copenhagen, Denmark

**Keywords:** Atropine, Myopia control, Spherical equivalent refraction, Axial length, Myopia, Eye drops

## Abstract

**Background:**

To investigate the efficacy and safety of 0.1% and 0.01% low-dose atropine eye drops in reducing myopia progression in Danish children.

**Methods:**

Investigator-initiated, placebo-controlled, double-masked, randomized clinical trial. Ninety-seven six- to twelve-year old myopic participants were randomized to 0.1% loading dose for six months followed by 0.01% for six months (loading dose group, Number (N) = 33), 0.01% for twelve months (0.01% group, N = 32) or vehicle for twelve months (placebo, N = 32). Primary outcomes were axial length and spherical equivalent refraction. Secondary outcomes included adverse events and reactions, choroidal thickness and ocular biometry. Outcomes were measured at baseline and three-month intervals. Data was analyzed with linear-mixed model analysis according to intention-to-treat.

**Results:**

Mean axial elongation was 0.10 mm less (95% confidence interval (CI): 0.17; 0.02, adjusted-p = 0.06) in the 0.1% loading dose and 0.07 mm less (95% CI: 0.15; 0.00, adjusted-p = 0.16) in the 0.01% group at twelve months compared to placebo. Mean spherical equivalent refraction progression was 0.24 D (95% CI: 0.05; 0.42) less in the loading dose and 0.19 D (95% CI: 0.00; 0.38) less in the 0.01% groups at twelve months, compared to placebo (adjusted-p = 0.06 and 0.14, respectively). A total of 108 adverse events were reported during the initial six-month loading dose period, primarily in the loading dose group, and 14 were reported in the six months following dose switching, all deemed mild except two serious adverse events, unrelated to the intervention.

**Conclusions:**

Low-dose atropine eye drops are safe over twelve months in otherwise healthy children. There may be a modest but clinically relevant reduction in myopia progression in Danish children after twelve months treatment, but the effect was statistically non-significant after multiple comparisons adjustment. After dose-switching at six months the loading dose group approached the 0.01% group, potentially indicating an early “rebound-effect”.

**Trial registration:**

this study was registered in the European Clinical Trials Database (EudraCT, number: 2018-001286-16) 05/11/2018 and first posted at www.clinicaltrials.gov (NCT03911271) 11/04/2019, prior to initiation.

**Supplementary Information:**

The online version contains supplementary material available at 10.1186/s12886-023-03177-9.

## Background

The prevalence of myopia has increased in recent years [1, 2], particularly in Asia, where up to 80% of school-age children are myopic [3]. In Denmark the prevalence is lower, with 18% of 12–13 year old children affected by myopia [4]. Myopia is most commonly a result of excessive ocular axial elongation during childhood, and high myopia predisposes to long-term complications such as retinal detachment, and myopic maculopathy [5, 6], compelling the search for therapeutic interventions to retard myopia progression.

Interventions to reduce myopia progression include changes to lifestyle. Increasing outdoor activities during elementary school recesses has been shown to delay myopia onset and reduce myopia progression [7–9]. It is not entirely clear why increased outdoor time has a protective effect on myopia, but some possible mechanisms are higher outdoor light intensity [10] or reduced peripheral retinal defocus in the outside environment, which might act as a stop-signal for further eye growth [11]. Increased amount of near-work might be associated with myopia progression and earlier myopia onset [12, 13], though the research does not uniformly show an association [13]. Optical interventions such as multifocal spectacle lenses [14], multifocal contact lenses [15], or rigid overnight-wear orthokeratology contact lenses [16] have also proven effective in reducing myopia progression, but contact lenses might not be the ideal intervention for all children, because of difficulties with handling and the, although rare, associated risk of infection [17]. Low-dose atropine eye drops is currently the most promising pharmacological intervention [18], and have in studies on Asian children been shown to slow myopia progression [19, 20]. The effect of low-dose atropine is dose-dependent [21]. However, due to a “rebound-effect” after eye drop-cessation, the Atropine for the Treatment of Myopia 2 (ATOM2) study reported that 0.01% ultimately had a superior efficacy, and a lower number of side effects compared to both 0.1% and 0.05% [22]. Similar efficacy of 0.01% has been confirmed in other studies [20, 23]. Notably the Low-Concentration Atropine for Myopia Progression (LAMP) study found 0.05% to be more efficacious compared to 0.01%, albeit with a significantly higher observed rate of side effects (photophobia) [20].

Myopia progression varies with ethnicity and social setting [24], and differences in sensitivity to atropine, and thereby side effects, could exist between ethnicities, for example due to variability in iris pigmentation [25]. While the efficacy and safety profile of low-dose atropine has been well-documented in Asian children, the intervention has been less examined, and is more controversial, in Caucasian children [26–28]. Our six-month interim analysis indicated similar early results in a Caucasian population as that observed in an Asian population [26]. In contrast, The Myopia Outcome Study of Atropine in Children (MOSAIC) found no significant difference between their 0.01% and placebo group on spherical equivalent refraction (SER) at two-year follow-up, but did find a small, significant effect on axial elongation [27]. They additionally subdivided their cohort based on ethnicity and found that the two-year efficacy was significant for both axial length (AL) and SER in participants of White ethnicity, but not in children of non-White ethnicity [27]. Similarly, The Western Australia ATOM (WA-ATOM)-study published results examining the effect in a multi-racial cohort at two-year follow-up and found a small, significant effect during the first 18 months of the intervention which did not retain significance at two-year follow-up [28]. They speculated that attrition bias and an older mean baseline age in the placebo group could have contributed to their non-significant results at two-year follow-up [28]. While the WA-ATOM study was not powered to detect differences in efficacy between racial groups, they found a smaller annual progression change in their cohort of Asian children compared to that reported in LAMP [28, 29], highlighting the potential important role of social setting.

Ultimately, questions remain about transferability of results between different ethnicities and social settings, what the optimal dosing regimen is [30], and the fact that treatment effects might not be sustained following treatment cessation.

In this study we investigated the safety and efficacy of one-year treatment with low-dose atropine eye drops for reducing myopia progression in six- to twelve-year old Danish children. Additionally, we wanted to see if a 0.1% loading dose for the first six months lead to a greater sustained effect.

## Materials and methods

The study was an investigator-initiated, placebo-controlled, double-masked, randomized clinical trial investigating the efficacy and safety of low-dose atropine eye drops in Danish myopic children. Details of the study design and results after the first six months have been published previously [26].

### Study Population

Six- to twelve-year old myopic children were recruited from ophthalmologists and optometrists across Denmark.

#### Inclusion criteria

Inclusion criterion for children aged six to nine years was spherical power of ≤ − 1 diopter (D) in at least one eye. Inclusion criterion for children aged nine to twelve years was spherical power of ≤−2 D in at least one eye. The higher ≤ − 2 D criterion for nine- to twelve-year-old children was chosen to ensure myopia progression for all participants, since we had no way of retrieving certain data about prior progression rates. For both age groups, maximum allowed astigmatism at inclusion was less than − 1.5 D.

#### Exclusion criteria

Exclusion criteria were myopia secondary to retinal dystrophies, collagenopathies (specifically Ehlers-Danlos, Marfan and Sticklers syndromes), other ocular pathologies, previous eye surgery, previous use of potential myopia prophylactic agents (e.g. 7-methylxanthine, atropine, orthokeratology, pirenzepine), non-compliance to eye examinations, serious systemic health issues or developmental disorders or delays.

#### Settings

Participants attended regular three-month visits at one of three research facilities located at the Department of Ophthalmology at Aarhus University Hospital, University Hospital of Southern Denmark - Vejle Hospital or Copenhagen University Hospital - Rigshospitalet-Glostrup.

### Interventions

Participants were randomized 1:1:1 by computer algorithm to 0.01% low-dose atropine eye drops for 24 months (0.01% group) vs. 0.1% loading dose for six months followed by 0.01% for 18 months (0.1% loading dose group) vs. vehicle eye drops for two years (placebo). Eye drops were applied nightly at bedtime in each eye. Compliance was evaluated using at-home administered checklists with boxes for marking daily trial medication use. Children who received eye drops for 75% of the intervention period were considered compliant. Before randomization an at-home administration of lubricating eye drops (Viskøse Øjendråber “Ophtha”, Hypromellose 3.5 mg/mL, Actavis Group PTC ehf., Hafnarfjordur, Iceland) were offered to potential participants to assess if they could comply with the study intervention. Photochromatic or near-addition glasses were reimbursed in cases of atropine-induced photophobia or near vision difficulties. The study is ongoing, and the 2-year intervention will be followed by a one-year washout period.

### Outcomes

Primary outcome measures were myopia progression as defined by axial length (AL) measured in non-cycloplegia and SER measured in cycloplegia. Secondary outcome measures included were adverse events and reactions (AE/AR), changes in choroidal thickness and ocular biometry (i.e., keratometry, anterior chamber depth (ACD), lens thickness, vitreous chamber depth) after one year of treatment.

### Sample size and power calculation

The power calculation was based on the progression in SER in myopic Danish school children [31]. To detect a 50% reduction in progression 36 months after initiating treatment, compared to placebo, with a significance level of 0.05 and a power of 80%, a sample size in each intervention group of minimum 21 participants was needed. Additional participants were recruited to account for the study length, drop-out, and an unknown effect size of low-dose atropine in non-Asian children.

### Randomization Procedure

The randomization was performed using an in-built computer algorithm in Research Electronic Data Capture (REDCap) [32] hosted at Capital Region, Denmark, which also contained our electronic clinical report form. The algorithm was based on a list of randomly created numbers with each assigned to a specific treatment. Allocation concealment was accomplished by masking parents, participants, and trial staff to randomization status. Statistical analysis was performed masked to randomization status.

### Examinations

Participants were examined at the screening-, baseline-, 3-, 6-, 9- and 12-month visits. Best-corrected visual acuity (BCVA) was measured using the HOTV chart (Precision Vision, La Salle, IL, USA) at near and distance (40 cm and 4 m). Amplitude of accommodation was measured using a Royal Air Force near point ruler using best-corrected distance spectacles. Autorefraction (Right group, Retinomax K-plus 3, Tokyo, Japan) was performed in non-cycloplegia and cycloplegia (by twice-applied cyclopentolate 1% eye drops (Minims Cyclopentolate Hydrochloride 1%, Bausch & Lomb Nordic AB, Stockholm, Sweden) five minutes apart followed by a 30-minute wait). SER was calculated as half the cylindrical refraction added to the spherical refraction. Push-plus subjective refraction was performed using autorefraction and current prescription as starting points. AL, ACD, central corneal thickness (CCT) and lens thickness were measured by optical biometry (IOLMaster 700, Carl Zeiss AG, Oberkochen, Germany). Iridocorneal angle was determined by Scheimflug imaging (Oculus GmbH, Pentacam HR System, Wetzlar, Germany). Sub-foveal choroidal thickness was determined by swept source optical coherence tomography (OCT, Topcon Europe Medical BV, Capelle aan den Ijssel, The Netherlands), before administration of dilating eye drops. The choroid was automatically segmented by the Topcon Automated Boundary Software (Topcon Europe Medical BV, The Netherlands) and afterwards reviewed by experienced observers who corrected any lingering mis-segmentation manually. Of the nine sectors automatically generated by the software, only the sub-foveal choroidal thickness was analyzed. Intra-ocular pressure (IOP) was measured by a rebound tonometer (iCare USA, iCare, Raleigh, North Carolina, United States) as the mean of five measurements. Pupil diameter was measured as the mean of five measurements under mesopic (4 lx) and photopic (300 lx) light intensities by pupillometry (DP-2000 Pupillometer, NeurOptics, CA, USA). Participants were asked at each visit whether they experienced any side effects including visual, ocular, peri-ocular or systemic anti-cholinergic side effects. Participants were specifically questioned about photophobia and blurred vision for distance and near. Ocular side effects questioned included eye redness/irritation, itching, pain, changes in lacrimal production and allergic reactions. Side effects from the eye-surroundings questioned were skin-changes, itching, edema or dryness. Systemic anti-cholinergic side effects questioned included dry skin, dry mouth or throat, facial redness, gastrointestinal symptoms, urinary retention or tachycardia.

### Statistical analysis

Linear mixed models were constructed with treatment and research facility as fixed effects using the R statistical software version 4.1.0 (R Program for Statistical Computing, Vienna, Austria) [33] and the LMMstar statistical package [34]. To account for the correlation in the repeated measurements, possible variance heterogeneity over time, and correlation between measurements obtained at the same facility, we assumed an unstructured covariance pattern. All ocular parameters were reported as the average of both eyes. For the statistical analysis, baseline values were assumed to be equal between intervention groups. Data were analyzed according to the intention-to-treat method. P-values were adjusted for multiple testing using the False Discovery Rate (FDR) [35]. Effect estimates with an adjusted-p-value < 0.05 were considered statistically significant.

## Results

**Fig. 1:**
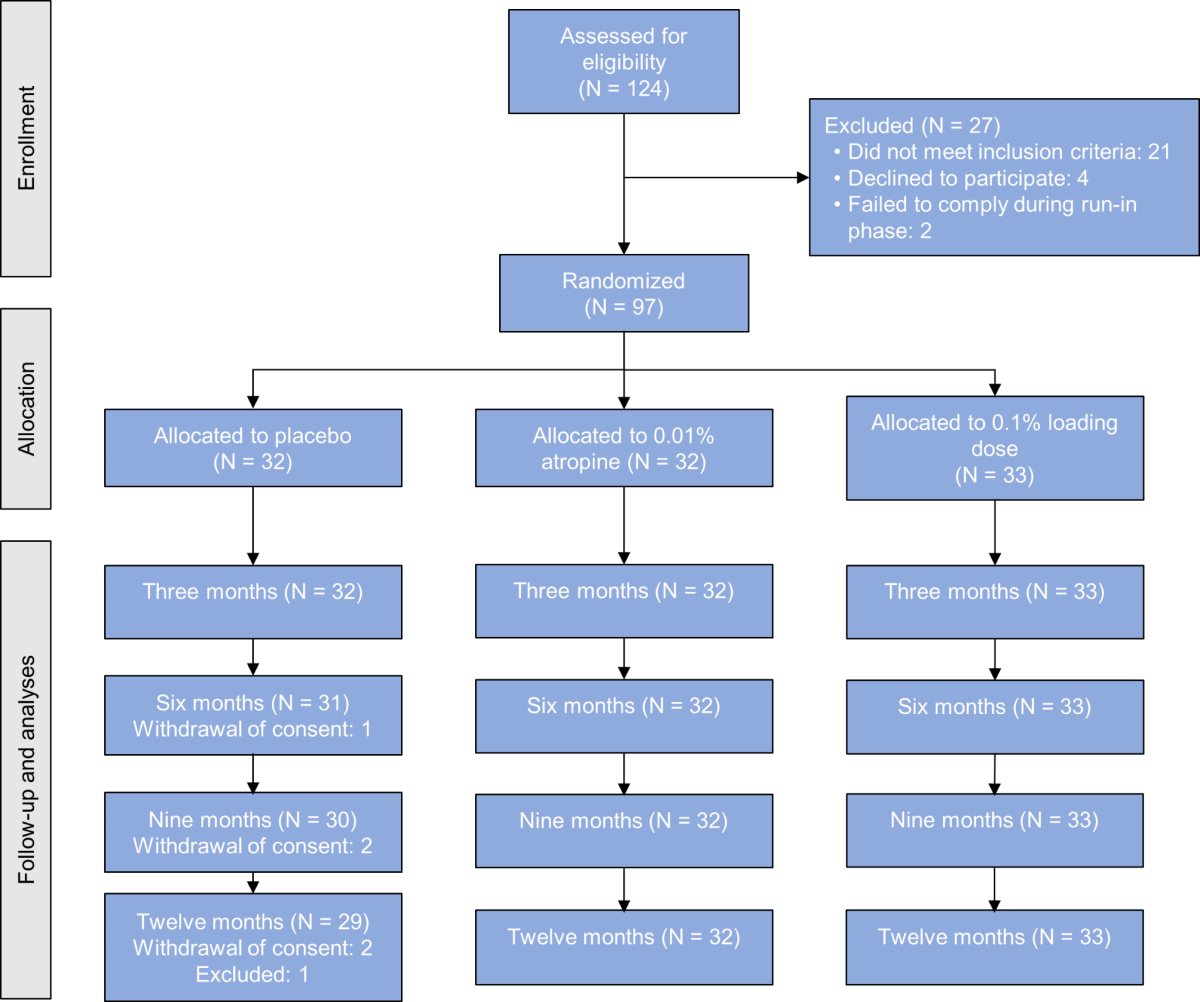
Consolidated Standards of Reporting Trials (CONSORT) flow-chart diagram of the study. Abbreviations: 0.1% loading dose, group receiving 0.1% for the first six months followed by 0.01% for 18 months; 0.01%, group receiving 0.01% for 24 months; N, Number

A total of 124 candidates were screened for eligibility (Fig. 1). Sixteen did not meet inclusion criteria, six did not want to participate in the study following the screening visit, three could not comply with the examinations and two could not comply with using eye drops. Thus, 97 participants were enrolled in the study and randomized to one of the three intervention groups. At the baseline visit the mean age of participants was 9.4 years (range 6–12), 43% were males, 59% of participants had blue eyes, 31% had brown eyes and 10% had green eyes. Mean baseline AL and SER was comparable across all groups. Mean baseline AL was 24.54 mm (standard deviation (SD): 0.90), 24.60 mm (SD: 0.86) and 24.68 (SD: 0.78) for the placebo, 0.1% loading dose and 0.01% group, respectively. Mean baseline SER was − 3.04 D (SD: 1.04), -2.97 (SD: 1.59) and − 2.94 (SD: 1.13) for the placebo, 0.1% loading dose and 0.01% group, respectively. Three (3%) participants were excluded from the trial before the twelve-month visit: Two participants withdrew consent and one participant wanted to try another myopia control method. Thus, 94 (97%) participants completed the twelve-month visit, 33 in the 0.1% loading dose group, 32 in the 0.01% group and 29 in the placebo group. All participants except one reported using the drops at least six times a week (i.e., above 75% compliance rate) at all visits.

### Change in axial length and spherical equivalent refraction after twelve months

**Fig. 2:**
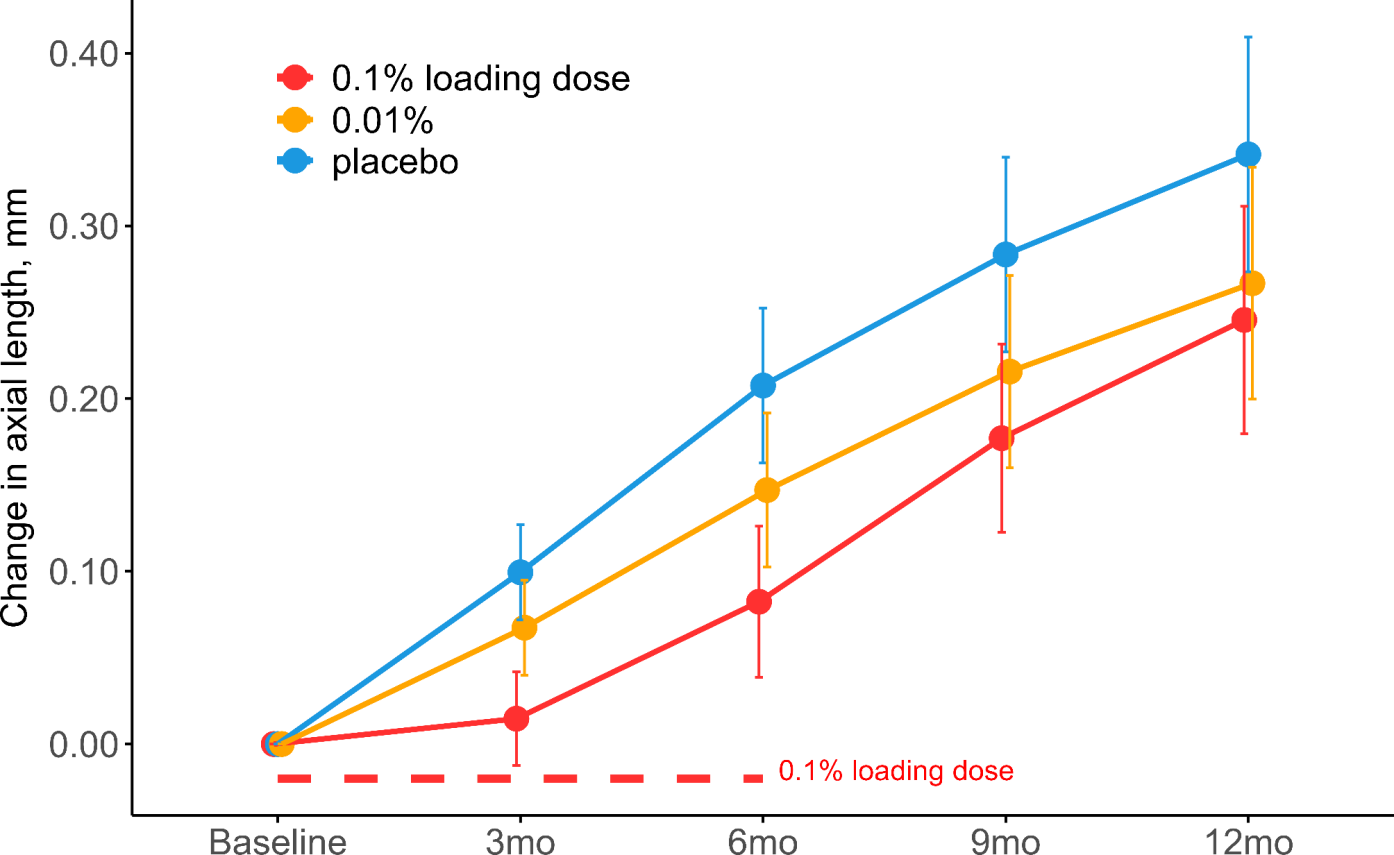
Estimated mean change in Axial Length pr. visit divided by intervention group. Error bars denote the 95% CI of the mean changes. Abbreviations: 0.1% loading dose, group receiving 0.1% for the first six months followed by 0.01% for 18 months; 0.01%, group receiving 0.01% for 24 months; mm, millimeters; mo, month; placebo, group receiving vehicle eye drops for 24 months

**Fig. 3:**
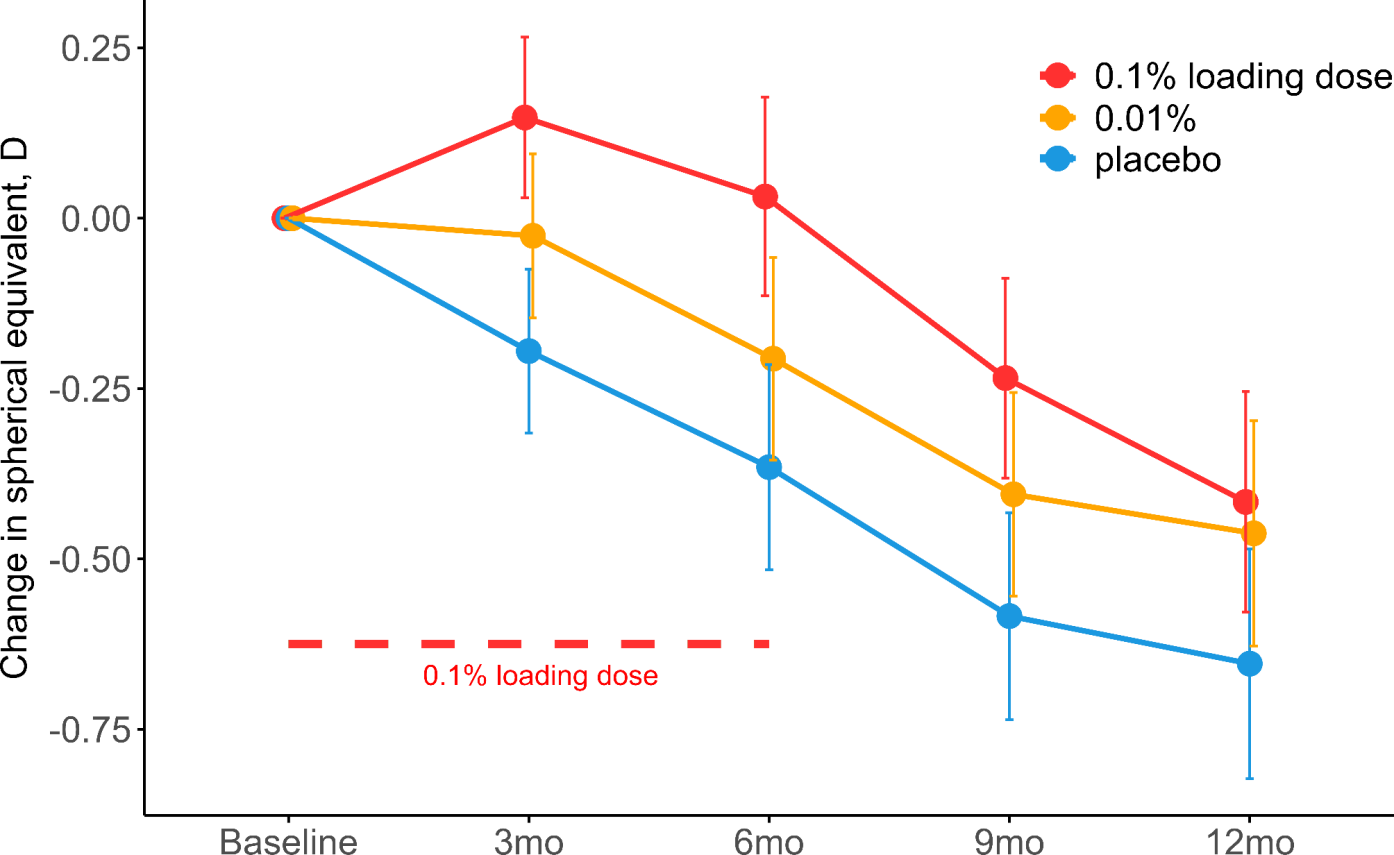
Estimated mean change in Spherical Equivalent Refraction pr. visit divided by intervention group. Error bars denote the 95% CI of the mean changes. Abbreviations: 0.1% loading dose, group receiving 0.1% for the first six months followed by 0.01% for 18 months; 0.01%, group receiving 0.01% for 24 months; mm, millimeters; mo, month; placebo, group receiving vehicle eye drops for 24 months; D, diopters; mo, month; placebo, group receiving vehicle eye drops for 24 months

At the twelve-month visit, AL had elongated 0.10 mm less (95% confidence interval (CI): 0.17; 0.02) in the group receiving 0.1% loading dose for the initial six months and 0.07 mm less (95% CI: 0.15; 0.00) in the 0.01% group, compared to placebo (Table 1; Fig. 2), but the effects were not statistically significant following multiple comparisons adjustment (adj-P = 0.06 and 0.16, respectively). At the twelve-month visit, SER had progressed by 0.24 D (95% CI: 0.05; 0.42) and 0.19 D (95% CI: 0.00; 0.38) less in the 0.1% loading dose and 0.01% groups respectively, but the effects were not statistically significant after multiple comparisons adjustment (adj-P = 0.06 and 0.14, respectively). The mean SER at 12-month follow-up was − 3.40 D (95% CI: −3.86; −2.95) and − 3.45 D (95% CI: −3.90; −2.99) in the 0.1% loading dose and 0.01% groups respectively, compared to − 3.64 D (95% CI: −4.10; −3.18) in the placebo group (Table 1; Fig. 3).

**Table 1:** Linear Mixed Model Effect Estimates of Treatment Group on Ocular Parameters

Time point\Group	Placebo	0.1% loading dose^a^	0.01%^b^
AL, mm			
Baseline	24.60 (24.35; 24.86)		
9-mo	24.88 (24.56; 25.20)	−0.11 (− 0.17; −0.04)	−0.07 (− 0.13; 0.00)
12-mo	24.94 (24.62; 25.26)	−0.10 (− 0.17; −0.02)	−0.07 (− 0.15; 0.00)
*12-mo adjusted-p*		*0.06*	*0.16*
**SER, diopters**			
Baseline	−2.99 (− 3.37; −2.60)		
9-mo	−3.57 (− 4.11; −3.03)	0.35 (0.17; 0.52)	0.18 (0.00; 0.35)
12-mo	−3.64 (− 4.19; −3.09)	0.24 (0.05; 0.42)	0.19 (0.00; 0.38)
*12-mo adjusted-p*		*0.06*	*0.14*
**IOP, mmHg**			
Baseline	16.1 (15.3; 17.0)		
9-mo	16.7 (14.6; 18.9)	-0.3 (-1.8; 1.2)	-0.7 (-2.2; 0.8)
12-mo	16.6 (14.5; 18.8)	0.2 (-1.3; 1.7)	0.0 (-1.5; 1.5)
*12-mo adjusted-p*		*0.88*	*0.98*
**Distance BCVA, LogMAR**			
Baseline	−0.10 (− 0.12; −0.09)		
9-mo	−0.11 (− 0.15; −0.07)	−0.01 (− 0.03; 0.01)	0.01 (− 0.01; 0.04)
12-mo	−0.12 (− 0.16; −0.08)	−0.02 (− 0.04; 0.01)	−0.00 (− 0.03; 0.02)
*12-mo adjusted-p*		*0.42*	*0.88*
**Near BCVA, LogMAR**			
Baseline	−0.07 (− 0.09; −0.05)		
9-mo	−0.06 (− 0.12; −0.01)	−0.02 (− 0.06; 0.02)	0.00 (− 0.04; 0.04)
12-mo	−0.09 (− 0.14; −0.04)	−0.01 (− 0.04; 0.02)	0.01 (− 0.02; 0.04)
*12-mo adjusted-p*		*0.78*	*0.52*
**Accommodation amplitude, diopters**			
Baseline	16.4 (15.3; 17.5)		
9-mo	15.4 (12.9; 17.0)	−0.8 (− 2.2; 0.6)	−0.2 (− 1.6; 1.2)
12-mo	16.1 (14.2; 19.1)	−0.7 (− 2.2; 0.8)	−1.0 (− 2.5; 0.5)
*12-mo adjusted-p*		*0.52*	*0.32*
**Mesopic pupil diameter, mm**			
Baseline	4.14 (3.92; 4.36)		
9-mo	4.07 (3.59; 4.55)	0.31 (0.01; 0.62)	0.54 (0.24; 0.85)
12-mo	4.10 (3.62; 4.57)	0.43 (0.13; 0.72)	0.51 (0.21; 0.80)
*12-mo adjusted-p*		0.02*	0.006*
**Photopic pupil diameter, mm**			
Baseline	2.71 (2.61; 2.81)		
9-mo	2.67 (2.46; 2.89)	0.21 (0.08; 0.35)	0.23 (0.10; 0.36)
12-mo	2.67 (2.45; 2.89)	0.18 (0.04; 0.33)	0.22 (0.08; 0.36)
*12-mo adjusted-p*		0.05*	0.01*

*Effect estimates for the placebo group are total to the given time point while effect estimates for the intervention groups (0.1% loading dose and 0.01%) are differences from the placebo group at the given time point. The baseline effect estimates are means across all groups at baseline. Abbreviations: AL, axial length; BCVA, best-corrected visual acuity; mo, months; p, p-value; adjusted-p, p-value adjusted by False Discovery Rate; SER, Spherical equivalent refraction*

^***a***^ *Change in the 0.1% loading dose group compared to placebo at the given time point*

^***b***^ *Change in the 0.01% group compared to placebo at the given time point*

* Statistically significant below our adjusted-p cut-off of 0.05

### Change in Anterior Chamber depth and sub-foveal Choroidal Thickness after twelve months treatment

ACD was 0.03 mm (95% CI: 0.01; 0.05, adjusted-p (adj-p) = 0.01) deeper in the 0.1% loading dose group at the twelve-month visit compared to placebo (Supplementary Table 1). The CCT, iridocorneal angle and lens thickness were comparable between groups at the twelve-month visit. Sub-foveal choroidal thickness was comparable between all groups at the twelve-month visit.

### Side effects after twelve months treatment

Mean IOP was within normal limits and comparable between groups at the twelve-month visit (Table 1). Amplitude of accommodation, distance and near BCVA were similar to baseline and comparable between all groups at the twelve-month visit. Mean mesopic pupil diameter was 0.43 mm (95% CI: 0.13; 0.72) greater in the 0.1% loading dose group and 0.54 mm (95% CI: 0.24; 0.85) greater in the 0.01% group at the twelve-month visit, compared to placebo (adj-p = 0.02 and 0.006, respectively) but not different between the two intervention groups. The mean difference in photopic pupil diameter compared to placebo in the 0.1% loading dose group at the nine-month visit was comparable to that in the 0.01% group (0.21 mm (95% CI: 0.08; 0.35) vs. 0.23 mm (95% CI: 0.10; 0.36), respectively), in contrast to the significantly larger difference between intervention groups and placebo during the loading dose phase (1.82 mm (95% CI: 1.57; 2.08) vs. 0.19 mm (95% CI: -0.06; 0.45) at the six-month visit, respectively, Supplementary Table 1). Compared to placebo, mean photopic pupil diameter remained larger at the twelve-month visit by 0.18 mm (95% CI: 0.04; 0.33) in the 0.1% loading dose group and 0.22 mm (95% CI: 0.08; 0.36) in the 0.01% group (adj-p = 0.05 and 0.01, respectively).

In total 14 adverse events and reactions (AE/AR) were reported at the nine- and twelve-month visits (Table 2). All were deemed mild except two Serious Adverse Events judged to be unrelated to the study drug (a lymphadenectomy and a suspicion of meningitis, see Supplementary Materials). Most frequently reported AEs/ARs were photophobia (Number (N) = 2), blur during near-work (N = 4) and eye redness/irritation (N = 3).

**Table 2:** Adverse Events

Group	Event	3mo	6mo	9mo	12mo
**0.1% loading dose**	Total events, N/total N (%)	22/33 (66%)	15/33 (45%)	6/33 (18%)	2/33 (6%)
	Eye redness/irritation, N/total N (%)	3/33 (9%)	2/33 (6%)	0/33 (0%)	1/33 (3%)
	Photophobia, N/total N (%)	16/33 (48%)	11/33 (33%)	2/33 (6%)	0/33 (0%)
	Blurred near vision, N/total N (%)	18/33 (55%)	12/33 (36%)	1/33 (3%)	1/33 (3%)
	Blurred distance vision, N/total N (%)	0/33 (0%)	0/33 (0%)	0/33 (0%)	0/33 (0%)
	Other, N/total N (%)	3/33 (9%)	2/33 (6%)	2/33 (6%)	0/33 (0%)
	Dilated pupils, N/total N (%)	11/33 (33%)	7/33 (21%)	1/33 (3%)	0/33 (0%)
**0.01%**	Total events, N/total N (%)	8/32 (25%)	1/32 (3%)	1/32 (3%)	1/32 (3%)
	Eye redness/irritation, N/total N (%)	2/32 (6%)	1/32 (3%)	1/32 (3%)	0/32 (0%)
	Photophobia, N/total N (%)	3/32 (9%)	0/32 (0%)	0/32 (0%)	0/32 (0%)
	Blurred near vision, N/total N (%)	0/32 (0%)	1/32 (3%)	0/32 (0%)	1/32 (3%)
	Blurred distance vision, N/total N (%)	0/32 (0%)	0/32 (0%)	0/32 (0%)	0/32 (0%)
	Other, N/total N (%)	5/32 (16%)	0/32 (0%)	0/32 (0%)	1/32 (3%)
	Dilated pupils, N/total N (%)	0/32 (0%)	0/32 (0%)	0/32 (0%)	0/32 (0%)
**Placebo**	Total events, N/total N (%)	6/32 (19%)	4/31 (13%)	1/30 (3%)	1/29 (3%)
	Eye redness/irritation, N/total N (%)	2/32 (6%)	2/31 (6%)	1/30 (3%)	0/29 (0%)
	Photophobia, N/total N (%)	0/32 (0%)	0/31 (0%)	0/30 (0%)	0/29 (0%)
	Blurred near vision, N/total N (%)	0/32 (0%)	0/31 (0%)	0/30 (0%)	1/29 (3%)
	Blurred distance vision, N/total N (%)	0/32 (0%)	0/31 (0%)	0/30 (0%)	0/29 (0%)
	Other, N/total N (%)	4/32 (13%)	3/31 (10%)	0/30 (0%)	1/29 (3%)
	Dilated pupils, N/total N (%)	0/32 (0%)	0/31 (0%)	0/30 (0%)	0/29 (0%)

Table 2*legend: “Total events” refers to the number of participants with one or more adverse events. Abbreviations: N, Number*

## Discussion

We investigated the effect and safety of 0.01% and 0.1% loading dose atropine eye drops in reducing myopia progression in Danish children following twelve months treatment. We found a small effect on myopia progression, which was non-significant following multiple comparisons adjustment. We found a moderate frequency of adverse events, primarily in participants receiving 0.1% loading dose, followed by a low frequency of adverse events after dose-switching to 0.01%. Mesopic and photopic pupil diameter was significantly increased in both intervention groups compared to placebo, indicating that the eye drops had been used.

We observed a dose-dependent effect on AL and SER progression which was greater in the 0.1% loading dose group than the 0.01% group, during the initial six-month loading dose phase. We found a non-significant effect on AL progression in our 0.01% group after twelve months of treatment similar in size to that found in the LAMP study (− 0.07 mm and − 0.05 mm, respectively) [20] and also a non-significant, similar reductive effect on SER progression (0.19 D difference from placebo in our study vs. 0.22 D in LAMP). Our sample size was comparably smaller, resulting in wider confidence intervals for estimation of the population mean, and our larger observed effect could therefore be a result of our sample being a statistical outlier. The one-year reductive effect on AL progression was also comparable to that reported in WA-ATOM [28] and Wei et al. [23] (− 0.07 mm (95% CI: 0.00; −0.15) compared to − 0.08 mm (95% CI: −0.02; −0.14) and − 0.09 mm (95% CI: −0.03; −0.15), respectively), who both found a statistically significant reductive effect, but to our knowledge did not employ multiple comparisons adjustment. Further, Wei et al. [23] had a high loss to follow-up (30% vs. 3% in our study), particularly in the intervention group, risking attrition bias. AL overall increased slightly more in the 0.01% group in Yam et al. [20] following one year of treatment compared to ours (0.36 mm vs. 0.27 mm) - This is likely explained by ethnic and social differences in myopia phenotype [24] and younger age in the LAMP study (8.2 years vs. our 9.4 years), since myopia appears to progress faster between years 6 to 10 [36]. The WA-ATOM study further subdivided their multi-racial cohort, examining the annual ancestry-specific effect of 0.01% on AL progression and found a -0.10 mm reduction in those of European ancestry, similar to the effect estimate observed in our study [28]. Their study was not powered to examine differences between ethnicities [28]. The MOSAIC study examined a predominantly European cohort and found a significant reductive effect of 0.01% on both AL and SER progression in White participants after two years treatment, but the effect was not significant at one-year follow-up. A limitation of MOSAIC was COVID-19 public health restrictions during the intervention period, which might have impacted results [27]. Our one-year effect estimates for reduction of myopia progression with 0.01% atropine are also comparable to a recently published study on North American and European children [37] - their participants were slightly younger (8.9 years), with a wider age inclusion criteria (3 to 17 years) and more ethnically heterogenous compared to ours.

A rebound effect has been reported after atropine cessation which speculatively may be reduced by dose-tapering rather than complete cessation of atropine eye drops [22]. Our study was designed to test this hypothesis by including a loading dose. We observed a slightly larger effect of 0.1% atropine than 0.01%, although with an overlap of confidence intervals, but we also saw that the loading dose group approached the lower dose group after 12 months of treatment. It will be interesting to follow the effect of the loading dose in a longer perspective.

Approximately half of participants in the 0.1% loading dose group experienced pupil-related side effects during the loading dose phase (i.e., the initial six months in this group). The LAMP study speculated that less iris pigmentation might result in an increased occurrence of pupil-related side effects [38]. However, distance and near BCVA was normal and comparable between groups at all visits. Additionally, no participants in the 0.1% loading dose group requested the available photochromatic glasses or near-vision add, but this could be attributed to the short duration of the loading dose phase for this intervention group. Following dose reduction in the 0.1% loading dose group, pupil-size related side effects were uncommon in both intervention groups. This is likely because few participants hereafter exceeded the 3 mm photopic pupil size that has been reported to lead to side effects [29, 30, 39]. When the 0.1% loading dose group switched over to 0.01%, the mesopic and photopic pupil diameter also promptly decreased, implying that pupil-related side effects can quickly be reduced by dose-tapering.

A strength of this study was the double-masked, placebo-controlled, randomized clinical trial-setup and low drop-out rate. Also, we retained a placebo group receiving vehicle in contrast to for example the ATOM2 study [22]. The LAMP study also retained a placebo group, but this group was switched to the 0.05% in the second year of treatment [29]. A limitation is that these are interim results with the full effect first being apparent after three years. A potential limitation is that we had no way of retrieving myopia progression rates prior to inclusion. We therefore decided that nine- to twelve-year old participants should have a more negative spherical power at inclusion compared to six- to nine-year old participants to ensure myopia progression. To account for the large number of significance tests performed and therefore the increased likelihood of confirming a non-existent correlation (i.e., committing a type I error), we employed the FDR for our multiple comparisons-adjustment and reported the adjusted p-values. These are preliminary one-year results and the effect of the loading-dose at longer follow-up, and the third-year wash-out period, will first be apparent in the final analysis. There are no plans to terminate the study before conclusion of the wash-out period.

## Conclusions

In conclusion, low-dose atropine eye drops seem to exhibit similar effectiveness in Danish children to that reported in Asian children following twelve months of treatment, but the effect was not significant following multiple comparisons adjustment. Low-dose atropine eye drops are safe, with a moderate amount of side effects, which primarily occurred in the 0.1% loading dose group, and which were promptly reduced following switch-over to the 0.01% dose. Low-dose atropine eye drops may have a small, but clinically relevant effect at reducing myopia progression in Danish children. Side effects related to pupil-dilation should not be a major concern when prescribing 0.01% low-dose atropine eye drops for Caucasian children.

### Electronic supplementary material

Below is the link to the electronic supplementary material.

Supplementary Material 1

Supplementary Material 2

## Data Availability

The datasets generated and analyzed during the current study are available from the corresponding author on reasonable request.

## List of Abbreviations

ACD: Anterior chamber depth
AE/AR: Adverse Event/Adverse Reaction
ATOM2: Atropine for the Treatment of Myopia 2 (study)
AL: Axial length
BCVA: Best-corrected visual acuity
CCT: Central corneal thickness
CI: Confidence interval
EudraCT: European Union Drug Regulating Authorities Clinical Trials Database
GCP: Good Clinical Practice
IOP: Intraocular pressure
FDR: False discovery rate
K1: The flat corneal meridian
K2: The steep corneal meridian
LAMP: Low-Concentration Atropine for Myopia Progression (study)
LogMAR: Logarithm to the minimal angle of resolution
MOSAIC: The Myopia Outcome Study of Atropine in Children (MOSAIC)
N: Number
REDCap: Research Electronic Data Capture
SER: Spherical equivalent refraction
WA-ATOM: The Western Australia ATOM (WA-ATOM)-study
