# Supplementary material for: Safety and efficacy of 0.01% and 0.1% low-dose atropine eye drop regimens for reduction of myopia progression in Danish children: a randomized clinical trial examining one-year effect and safety
Source: BMC Ophthalmol. 2023 Oct 30;23:438. doi: 10.1186/s12886-023-03177-9 (PMC10614417; doi:10.1186/s12886-023-03177-9)
Supplement: Supplementary file 1 — Supplementary Material 1 [file 12886_2023_3177_MOESM1_ESM.docx]

**Specification of Serious Adverse Events**

One participant was hospitalized under suspicion of meningitis, the suspicion of which was later abated, one participant was hospitalized for an acute appendectomy and subsequently remitted fully, and one participant was hospitalized for a routine lymphadenectomy which pathology later confirmed to be benign. None of the SAEs were related to the intervention, i.e., there were no Serious Adverse Reactions (SARs). Further, there were no Suspected Unexpected Serious Adverse Reactions (SUSARs, i.e., there were no serious, unexpected adverse reactions that with reasonable possibility could be related to the eye drops regimen).
