# Supplementary material for: Safety and efficacy of 0.01% and 0.1% low-dose atropine eye drop regimens for reduction of myopia progression in Danish children: a randomized clinical trial examining one-year effect and safety
Source: BMC Ophthalmol. 2023 Oct 30;23:438. doi: 10.1186/s12886-023-03177-9 (PMC10614417; doi:10.1186/s12886-023-03177-9)
Supplement: Supplementary file 2 — Supplementary Material 2 [file 12886_2023_3177_MOESM2_ESM.docx]

Supplementary Table 1: All Linear Mixed Model Effect Estimates of Treatment Group on Ocular Parameters

| **Group**  **Time point** | **Placebo** | **0.1% loading dose^a^** | **0.01% ^b^** |
| --- | --- | --- | --- |
| **AL, mm** | | | |
| Baseline | 24.60 (24.35; 24.86) | | |
| 3-mo | 24.70 (24.42; 24.99) | -0.08 (-0.12; -0.05) | -0.03 (-0.06; 0.00) |
| *p* \| *adjusted-p* |  | *<0.001* \| *<0.001** | *0.05* \| *0.14* |
| 6-mo | 24.80 (24.51; 25.11) | -0.13 (-0.18; -0.07) | -0.06 (-0.11; -0.01) |
| *p* \| *adjusted-p* |  | *<0.001* \|*<0.001** | *0.02** \| *0.08* |
| 9-mo | 24.88 (24.56; 25.20) | -0.11 (-0.17; -0.04) | -0.07 (-0.13; 0.00) |
| *p* \| *adjusted-p* |  | *<0.01* \| *0.01** | *0.04* \| *0.13* |
| 12-mo | 24.94 (24.62; 25.26) | -0.10 (-0.17; -0.02) | -0.07 (-0.15; 0.00) |
| *p* \| *adjusted-p* |  | *0.02* \| *0.06* | *0.06* \| *0.16* |
| **SER, diopters** | | | |
| Baseline | -2.99 (-3.37; -2.60) | | |
| 3-mo | -3.19 (-3.68; -2.67) | 0.34 (0.20; 0.48) | 0.17 (0.03; 0.31) |
| *p* \| *adjusted-p* |  | *<0.001* \| *<0.001** | *0.02* \| *0.07* |
| 6-mo | -3.36 (-3.89; -2.81) | 0.40 (0.22; 0.57) | 0.16 (-0.02; 0.34) |
| *p* \| *adjusted-p* |  | *<0.001* \| *<0.001** | *0.07* \| *0.19* |
| 9-mo | -3.57 (-4.11; -3.03) | 0.35 (0.17; 0.52) | 0.18 (0.00; 0.35) |
| *p* \| *adjusted-p* |  | *<0.001*\| *0.001** | *0.05* \| *0.13* |
| 12-mo | -3.64 (-4.19; -3.09) | 0.24 (0.05; 0.42) | 0.19 (0.00; 0.38) |
| *p* \| *adjusted-p* |  | *0.02* \| *0.06* | *0.05* \| *0.14* |
| **Distance BCVA, LogMAR** | | | |
| Baseline | -0.10 (-0.12; -0.09) | | |
| 3-mo | -0.10 (-0.14; -0.06) | -0.04 (-0.06; -0.01) | 0.00 (-0.02; 0.02) |
| *p* \| *adjusted-p* |  | *<0.01* \| *0.01** | *0.92* \| *0.95* |
| 6-mo | -0.11 (-0.15; -0.07) | -0.01 (-0.03; 0.01) | 0.02 (-0.01; 0.04) |
| *p* \| *adjusted-p* |  | *0.46* \| *0.61* | *0.21* \| *0.38* |
| 9-mo | -0.11 (-0.15; -0.07) | -0.01 (-0.03; 0.01) | 0.01 (-0.01; 0.04) |
| *p* \| *adjusted-p* |  | *0.42* \| *0.57* | *0.27* \| *0.43* |
| 12-mo | -0.12 (-0.16; -0.08) | -0.02 (-0.04; 0.01) | -0.00 (-0.03; 0.02) |
| *p* \| *adjusted-p* |  | *0.25* \| *0.42* | *0.80* \| *0.88* |
| **Near BCVA, LogMAR** | | | |
| Baseline | -0.07 (-0.09; -0.05) | | |
| 3-mo | -0.09 (-0.14; -0.03) | 0.04 (-0.01; 0.07) | 0.03 (-0.01; 0.06) |
| *p* \| *adjusted-p* |  | *0.02** \| *0.08** | *0.11* \| *0.24* |
| 6-mo | -0.09 (-0.14; -0.03) | 0.04 (0.00; 0.07) | 0.03 (-0.01; 0.06) |
| *p* \| *adjusted-p* |  | *0.05* \| *0.14* | *0.17* \| *0.32* |
| 9-mo | -0.06 (-0.12; -0.01) | -0.02 (-0.06; 0.02) | 0.00 (-0.04; 0.04) |
| *p* \| *adjusted-p* |  | *0.29* \| *0.46* | *0.96* \| *0.97* |
| 12-mo | -0.09 (-0.14; -0.04) | -0.01 (-0.04; 0.02) | 0.01 (-0.02; 0.04) |
| *p* \| *adjusted-p* |  | *0.64* \| *0.78* | *0.36* \| *0.52* |
| **Accommodation amplitude, diopters** | | | |
| Baseline | 16.4 (15.3; 17.5) | | |
| 3-mo | 14.8 (12.2; 17.4) | -5.7 (-7.3; -4.1) | -1.1 (-2.7; 0.5) |
| *p* \| *adjusted-p* |  | *<0.001* \| *<0.001** | *0.19* \| *0.34* |
| 6-mo | 14.3 (11.7; 16.9) | -4.9 (-6.4; -3.4) | -0.4 (-1.9; 1.1) |
| *p* \| *adjusted-p* |  | *<0.001* \| *<0.001** | *0.56* \| *0.71* |
| 9-mo | 15.4 (12.9; 17.0) | -0.8 (-2.2; 0.6) | -0.2 (-1.6; 1.2) |
| *p* \| *adjusted-p* |  | *0.26* \| *0.42* | *0.76* \| *0.85* |
| 12-mo | 16.1 (14.2; 19.1) | -0.7 (-2.2; 0.8) | -1.0 (-2.5; 0.5) |
| *p* \| *adjusted-p* |  | *0.35* \| *0.52* | *0.17* \| *0.32* |
| **IOP, mmHg** | | | |
| Baseline | 16.1 (15.3; 17.0) | | |
| 3-mo | 15.9 (13.8; 18.0) | 1.5 (0.2; 2.9) | 0.5 (-0.9; 1.8) |
| *p* \| *adjusted-p* |  | *0.03* \| *0.09* | *0.49* \| *0.64* |
| 6-mo | 16.8 (14.8; 18.9) | 0.1 (-1.2; 1.4) | -0.7 (-2.0; 0.6) |
| *p* \| *adjusted-p* |  | *0.88* \| *0.94* | *0.26* \| *0.42* |
| 9-mo | 16.7 (14.6; 18.9) | -0.3 (-1.8; 1.2) | -0.7 (-2.2; 0.8) |
| *p* \| *adjusted-p* |  | *0.71* \| *0.82* | *0.37* \| *0.53* |
| 12-mo | 16.6 (14.5; 18.8) | 0.2 (-1.3; 1.7) | 0.0 (-1.5; 1.5) |
| *p* \| *adjusted-p* |  | *0.80* \| *0.88* | *0.98* \| *0.98* |
| **Mesopic pupil diameter, mm** | | | |
| Baseline | 4.14 (3.92; 4.36) | | |
| 3-mo | 4.21 (3.69; 4.73) | 2.03 (1.68; 2.39) | 0.30 (-0.05; 0.65) |
| *p* \| *adjusted-p* |  | *<0.001* \| *<0.001** | *0.09* \| *0.22* |
| 6-mo | 4.07 (3.56; 4.13) | 2.18 (1.84; 2.52) | 0.42 (0.08; 0.76) |
| *p* \| *adjusted-p* |  | *<0.001** \| *<0.001** | *0.01** \| *0.06* |
| 9-mo | 4.07 (3.59; 4.55) | 0.31 (0.01; 0.62) | 0.54 (0.24; 0.85) |
| *p* \| *adjusted-p* |  | *0.05** \| *0.13* | *<0.001** \| *0.004** |
| 12-mo | 4.10 (3.62; 4.57) | 0.43 (0.13; 0.72) | 0.51 (0.21; 0.80) |
| *p* \| *adjusted-p* |  | *0.01** \| *0.02** | *<0.001** \| *0.006** |
| **Photopic pupil diameter, mm** | | | |
| Baseline | 2.71 (2.61; 2.81) | | |
| 3-mo | 2.76 (2.40; 3.11) | 1.83 (1.53; 2.13) | 0.17 (-0.13; 0.47) |
| *p* \| *adjusted-p* |  | *<0.001* \| *<0.001** | *0.26* \| *0.42* |
| 6-mo | 2.67 (2.35; 2.99) | 1.82 (1.57; 2.08) | 0.19 (-0.06; 0.45) |
| *p* \| *adjusted-p* |  | *<0.001* \| *<0.001** | *0.14* \| *0.28* |
| 9-mo | 2.67 (2.46; 2.89) | 0.21 (0.08; 0.35) | 0.23 (0.10; 0.36) |
| *p* \| *adjusted-p* |  | *<0.01* \| *0.01** | *<0.001* \| *0.006** |
| 12-mo | 2.67 (2.45; 2.89) | 0.18 (0.04; 0.33) | 0.22 (0.08; 0.36) |
| *p* \| *adjusted-p* |  | *0.01* \| *0.05** | *0.002* \| 0.01* |
| **Sub-foveal choroidal thickness, μm** | | | |
| Baseline | 242 (222; 262) | | |
| 3-mo | 243 (215; 269) | 14 (7; 22) | 3 (-4; 11) |
| *p* \| *adjusted-p* |  | *<0.001* \| *0.002** | *0.39* \| *0.55* |
| 6-mo | 239 (211; 268) | 13 (3; 23) | 2 (-8; 12) |
| *p* \| *adjusted-p* |  | *0.01* \| *0.05** | *0.70* \| *0.82* |
| 9-mo | 243 (215; 271) | 4 (-5; 13) | 4 (-5; 13) |
| *p* \| *adjusted-p* |  | 0.38 \| *0.54* | *0.36* \| *0.52* |
| 12-mo | 245 (217; 251) | 1 (-7; 10) | 4 (-5; 14) |
| *p* \| *adjusted-p* |  | 0.76 \| *0.85* | *0.33* \| *0.51* |
| **ACD, mm** | | | |
| Baseline | 3.30 (3.23; 3.37) | | |
| 3-mo | 3.30 (3.22; 3.39) | 0.05 (0.03; 0.07) | 0.01 (0.00; 0.03) |
| *p* \| *adjusted-p* |  | *<0.001* \| *<0.001** | *0.12* \| *0.26* |
| 6-mo | 3.31 (3.22; 3.40) | *0.05 (0.03; 0.07)* | 0.02 (-0.01; 0.04) |
| *p* \| *adjusted-p* |  | *<0.001* \| *<0.001** | *0.14* \| *0.28* |
| 9-mo | 3.31 (3.21; 3.41) | 0.02 (-0.01; 0.04) | 0.02 (-0.01; 0.05) |
| *p* \| *adjusted-p* |  | *0.13* \| *0.27* | *0.11* \| *0.25* |
| 12-mo | 3.31 (3.22; 3.40) | *0.03 (0.01; 0.05)* | 0.01 (-0.01; 0.03) |
| *p* \| *adjusted-p* |  | <0.01 \| *0.01** | *0.16* \| *0.31* |
| **Iridocorneal angle, degrees** | | | |
| Baseline | 43.9 (42.3; 45.6) | | |
| 3-mo | 44.6 (41.4; 47.8) | -4.1 (-5.9; -2.2) | -2.0 (-3.9; -0.2) |
| *p* \| *adjusted-p* |  | *<0.001* \| *<0.001** | *0.03* \| *0.10* |
| 6-mo | 45.4 (42.1; 48.7) | *-*4.1 (-6.0; -2.2) | -1.4 (-3.3; 0.5) |
| *p* \| *adjusted-p* |  | *<0.001* \| *<0.001** | *0.15* \| *0.31* |
| 9-mo | 44.6 (41.6; 47.6) | -1.2 (-2.8; 0.5) | -1.8 (-3.4; -0.2) |
| *p* \| *adjusted-p* |  | *0.16* \| *0.31* | *0.03* \| *0.10* |
| 12-mo | 45.1 (42.3; 48.0) | -1.4 (-2.8; 0.0) | -1.3 (-2.7; 0.2) |
| *p* \| *adjusted-p* |  | *0.05* \| *0.14* | *0.08* \| *0.20* |
| **CCT, μm** | | | |
| Baseline | 551 (542; 560) | | |
| 3-mo | 553 (542; 565) | -1 (-3; 1) | -2 (-5; 0) |
| *p* \| *adjusted-p* |  | *0.35* \| *0.52* | *0.06* \| *0.15* |
| 6-mo | 554 (542; 565) | 0 (-2; 3) | -2 (-4; 1) |
| *p* \| *adjusted-p* |  | *0.92* \| *0.95* | *0.15* \| *0.31* |
| 9-mo | 554 (542; 565) | -1 (-4; 1) | -1 (-4; 1) |
| *p* \| *adjusted-p* |  | *0.31* \| *0.49* | *0.35* \| *0.52* |
| 12-mo | 554 (543; 566) | 0 (-2; 3) | -1 (-4; 1) |
| *p* \| *adjusted-p* |  | *0.92* \| *0.95* | *0.23* \| *0.39* |
| **K1 (front), diopters** | | | |
| Baseline | 43.03 (42.59; 43.48) | | |
| 3-mo | 43.00 (42.52; 43.39) | 0.00 (-0.05; 0.05) | -0.01 (-0.06; 0.04) |
| *p* \| *adjusted-p* |  | *0.93* \| *0.95* | *0.66* \| *0.79* |
| 6-mo | 42.98 (42.50; 43.46) | 0.00 (-0.05; 0.05) | 0.01 (-0.03; 0.06) |
| *p* \| *adjusted-p* |  | *0.76* \| *0.85* | *0.61* \| *0.75* |
| 9-mo | 42.97 (42.45; 43.45) | -0.06 (-0.16; 0.03) | -0.02 (-0.11; 0.08) |
| *p* \| *adjusted-p* |  | *0.19* \| *0.34* | *0.71* \| *0.82* |
| 12-mo | 42.91 (42.42; 43.4) | 0.00 (-0.05; 0.06) | 0.02 (-0.04; 0.07) |
| *p* \| *adjusted-p* |  | *0.88* \| *0.94* | *0.53* \| *0.68* |
| **K2 (front), diopters** | | | |
| Baseline | 43.89 (43.43; 44.35) | | |
| 3-mo | 43.85 (43.42; 44.34) | 0.10 (0.04; 0.17) | 0.00 (-0.06; 0.07) |
| *p* \| *adjusted-p* |  | *0.002* \| *0.01** | *0.96* \| *0.97* |
| 6-mo | 43.89 (43.36; 44.40) | 0.03 (-0.05; 0.11) | 0.02 (-0.06; 0.09) |
| *p* \| *adjusted-p* |  | *0.45* \| 0.60 | *0.68* \| *0.81* |
| 9-mo | 43.87 (43.36; 44.38) | 0.02 (-0.05; 0.08) | 0.01 (-0.05; 0.08) |
| *p* \| *adjusted-p* |  | *0.61* \| *0.75* | *0.71* \| *0.82* |
| 12-mo | 43.85 (43.33; 44.35) | 0.06 (-0.01; 0.12) | 0.03 (-0.04; 0.09) |
| *p* \| *adjusted-p* |  | *0.09* \| *0.21* | *0.41* \| *0.57* |
| **Lens thickness, mm** | | | |
| Baseline | 3.32 (3.28; 3.38) | | |
| 3-mo | 3.32 (3.27; 3.39) | -0.03 (-0.04; -0.01) | -0.01 (-0.03; 0.01) |
| *p* \| *adjusted-p* |  | *0.0*05 \| *0.02** | *0.16* \| *0.31* |
| 6-mo | 3.32 (3.25; 3.38) | -0.02 (-0.04; -0.00) | -0.01 (-0.03; 0.01) |
| *p* \| *adjusted-p* |  | *0.02* \| *0.06* | *0.34* \| *0.52* |
| 9-mo | 3.32 (3.25; 3.40) | 0.00 (-0.03; 0.02) | -0.01 (-0.03; 0.02) |
| *p* \| *adjusted-p* |  | *0.84* \| 0.91 | *0.59* \| *0.73* |
| 12-mo | 3.33 (3.27; 3.40) | -0.01 (-0.03; 0.01) | -0.01 (-0.03; 0.01) |
| *p* \| *adjusted-p* |  | *0.55* \| *0.70* | *0.42* \| *0.57* |

Effect estimates for the placebo group are total to the given time point while effect estimates for the intervention groups (0.1% loading dose and 0.01%) are differences from the placebo group at the given time point. Abbreviations: ACD, anterior chamber depth; AL, axial length; BCVA, best-corrected visual acuity; CCT, central corneal thickness; IOP, intra-ocular pressure; K1, the flat corneal meridian; K2, the steep corneal meridian; mo, months; p, p-value; adjusted-p, p-value adjusted by False Discovery Rate; SER, Spherical equivalent refraction.

**^a^** Change in the 0.1% loading dose group compared to placebo at the given time point.

**^b^** Change in the 0.01% group compared to placebo at the given time point.

** Statistically significant below our adjusted-p cut-off of 0.05*
